# Somatostatin Ameliorates β-Amyloid-Induced Cytotoxicity via the Regulation of CRMP2 Phosphorylation and Calcium Homeostasis in SH-SY5Y Cells

**DOI:** 10.3390/biomedicines9010027

**Published:** 2021-01-02

**Authors:** Seungil Paik, Rishi K. Somvanshi, Helen A. Oliveira, Shenglong Zou, Ujendra Kumar

**Affiliations:** Faculty of Pharmaceutical Sciences, The University of British Columbia, Vancouver, BC V6T 1Z3, Canada; paikseungil@gmail.com (S.P.); rishiks@mail.ubc.ca (R.K.S.); aophelen@gmail.com (H.A.O.); zoe.s.long@gmail.com (S.Z.)

**Keywords:** β-Amyloid, calpain, Collapsin Response Mediator Protein-2, human-neuroblastoma SH-SY5Y cells, Somatostatin-14, somatostatin receptor

## Abstract

Somatostatin is involved in the regulation of multiple signaling pathways and affords neuroprotection in response to neurotoxins. In the present study, we investigated the role of Somatostatin-14 (SST) in cell viability and the regulation of phosphorylation of Collapsin Response Mediator Protein 2 (CRMP2) (Ser522) via the blockade of Ca^2+^ accumulation, along with the inhibition of cyclin-dependent kinase 5 (CDK5) and Calpain activation in differentiated SH-SY5Y cells. Cell Viability and Caspase 3/7 assays suggest that the presence of SST ameliorates mitochondrial stability and cell survival pathways while augmenting pro-apoptotic pathways activated by Aβ. SST inhibits the phosphorylation of CRMP2 at Ser522 site, which is primarily activated by CDK5. Furthermore, SST effectively regulates Ca^2+^ influx in the presence of Aβ, directly affecting the activity of calpain in differentiated SH-SY5Y cells. We also demonstrated that SSTR2 mediates the protective effects of SST. In conclusion, our results highlight the regulatory role of SST in intracellular Ca^2+^ homeostasis. The neuroprotective role of SST via axonal regeneration and synaptic integrity is corroborated by regulating changes in CRMP2; however, SST-mediated changes in the blockade of Ca^2+^ influx, calpain expression, and toxicity did not correlate with CDK5 expression and p35/25 accumulation. To summarize, our findings suggest two independent mechanisms by which SST mediates neuroprotection and confirms the therapeutic implications of SST in AD as well as in other neurodegenerative diseases where the effective regulation of calcium homeostasis is required for a better prognosis.

## 1. Introduction

Alzheimer’s disease (AD) is a progressive neurodegenerative disorder and the most common form of dementia in the elderly population. Standard clinical features of the disease include memory loss, abnormal social behavior, and deterioration of cognitive function [1,2,3]. AD is characterized by the formation of amyloid plaques, composed of abnormally truncated fragments of the amyloid precursor protein called β-amyloid (Aβ), and intracellular neurofibrillary tangles (NFT), consisting of hyperphosphorylated Tau protein [4,5]. The complex pathophysiology observed in AD is associated with the accumulation of plaques and the formation of NFTs, along with other pathological changes, resulting in synaptic dysfunction, excitotoxicity, dendritic spine loss and overall destabilization of the neural network [6,7]. The overaccumulation of Aβ is considered as the prominent cause of disease severity and neuronal cell death; however, the precise mechanism of interconnecting AD onset and progression is not fully understood, despite the identification of signaling pathways that exert determinant roles [8,9]. One such crucial signaling molecule that may represent a critical determinant is collapse response mediator 2 (CRMP2). Initially identified as a signaling molecule of a repulsive axon growth and guidance molecule Semaphorin3A, CRMP2 has since been identified as a critical marker of synapse formation, the establishment of neuronal cell polarity, dendritic patterning, learning, and memory [10,11]. In particular, CRMP2 regulates neuronal microtubule dynamics by binding to the tubulin heterodimers, leading to polymerization, in addition to colocalization and binding to actin [12,13,14,15]. Furthermore, CRMP2 also plays a critical role in the transportation of soluble tubulin and vesicles by acting as a cargo adaptor protein [16,17].

Like many other microtubule-binding proteins, such as Tau or microtubule-associated proteins (MAP), CRMP2 is phosphorylated by cyclin-dependent kinase (CDK5) and glycogen synthase kinase-3β (GSK-3β) near its C-terminus. Specifically, CDK5-mediated phosphorylation of CRMP2 at Ser522 primes the subsequent phosphorylation by GSK-3β at sites Ser518, Thr514 and Thr509 [18,19,20,21]. In addition to Cdk5 and GSK-3β, Rho/Rho-associated protein kinase has also been identified to phosphorylate CRMP2 at Thr555 [11]. Taken together, the phosphorylation of CRMP2 at these sites is associated with the regulation of neurite outgrowth, possibly due to the modifications of microtubule dynamics [19,21]. Several previous studies have reported hyperphosphorylation of CRMP2 in AD patients when compared to the age-matched control [2,19,20]. However, the exact mechanism of CRMP2 phosphorylation during the progression of AD remains elusive. Although controversy exists, it is well established that the hyperphosphorylation of CRMP2 occurs before the onset of pathology in the AD mouse model, implicating CRMP2 hyperphosphorylation as an early indicator of AD [2].

CDK5 is the primary kinase responsible for the CRMP2 phosphorylation at Ser522 [22,23]. CDK5 plays a critical role in the CNS, including neuronal migration, synapse formation, plasticity, and neurogenesis [24,25,26,27,28,29]. In contrast to other members of the CDK family that are regulated by p21 and p27, CDK5 activity is mainly regulated by p35 [30,31]. Moreover, while the activation of CDK5 by p35 in the physiological condition is essential for normal neuronal development, synaptic activity, and axonal transport, the abnormal activation of CDK5 leads to cell death and neurodegeneration [24,26,32,33,34,35,36,37]. In AD, the abnormal increase in CDK5 activation leading to hyperphosphorylation of various tubulin-associated proteins, including Tau and CRMP2, is associated with the accumulation of truncated fragments of p35 called p25, which induces the constitutive activation and mislocalization of CDK5 in vivo [37]. In this regard, the same study has also determined that calpain mediates the cleavage of p35 into p25 [37]. Calpain is a crucial enzyme involved in calcium-mediated neurodegeneration [38]. In AD, the accumulation of Aβ leads to the increase in intracellular Ca^2+^ levels, mitochondrial Ca^2+^ overload, production of pro-apoptotic proteins such as cytochrome *c*, and generation of superoxide radicals, eventually resulting in cell death and neurodegeneration [39]. We have previously demonstrated the effect of SST in promoting the retinoic acid (RA)-induced differentiation of SH-SY5Y cells [40]. We hypothesize that the identification of a molecule capable of downregulating the hyperphosphorylation of CRMP2 via the blockade of Ca^2+^ accumulation in AD may serve as a novel therapeutic agent.

We recently demonstrated that Somatostatin-14 (SST) mediates the promotion of the overall neurite length in RA-differentiated SH-SY5Y cells, with specific effects on microtubule-associated proteins such as MAP2 and Tau [40]. CRMP2 is a microtubule-associated protein and exhibits a close resemblance with MAP2 and Tau, with significant changes during the progression of AD. Taking this into consideration, we hypothesize that SST might be involved in the regulation of CRMP2 during the differentiation of SH-SY5Y cells. Among the various phosphorylation sites of CRMP2, we have focused on the Ser522 site due to its dual role, first as a phosphorylation site and second as the requirement in phosphorylation of a subsequent site, Thr514. Accordingly, in the present study, we sought to determine the role of SST and a possible mechanism involving the phosphorylation of Ser522 in the presence of Aβ_1-42_-induced toxicity in SH-SY5Y cells as an in-vitro model of AD. Our results revealed SST as a novel molecule capable of inhibiting the Aβ-induced hyper-influx of Ca^2+^, leading to the inhibition of calpain activity. Furthermore, SST inhibits the p35/p25-induced hyper-activation of CDK5 and the subsequent hyper-phosphorylation of CRMP2.

## 2. Experimental Section

### 2.1. SH-SY5Y Cell Culture

Human SH-SY5Y neuroblastoma cells were kindly obtained from Dr. Neil Cashman, University of British Columbia, BC, Canada, and grown as described earlier [40]. Briefly, the cells were grown on a 75 cm^2^ culture flask coated with Matrigel (10 mg/mL, BD Bioscience, San Jose, CA, USA). The culture medium comprised Dulbecco’s Modified Eagles Medium (DMEM; Invitrogen, Burlington, ON, Canada) supplemented with 10% fetal bovine serum (FBS), penicillin (100 U/mL) and streptomycin (100 μg/mL) in a 5% CO_2_ humidified incubator at 37 °C. For neuronal differentiation, the cells were treated with all-trans-retinoic acid (RA, 10 μM, Sigma, St. Louis, MO, USA) for 5–7 days as previously described [41]. All experiments were performed on cells differentiated for 5–7 days unless otherwise stated. Treatments with Aβ_1-42_ (Anaspec, Fremont, CA, USA) or SST-14 (Bachem, Torrance, CA, USA) were performed as described in the methods.

### 2.2. MTT Cell Viability Assay

To determine cell viability in response to Aβ, SH-SY5Y cells were processed for the MTT (3-(4,5-dimethylthiazol-2-yl)-2,5-diphenyl tetrazolium bromide) assay, as previously described [42]. Briefly, differentiated SH-SY5Y cells were treated with increasing concentrations of Aβ_1-42_ (0, 1, 5, 10 and 20 μM) or SST (0.4, 2 and 10 μM) alone, and with the combination of Aβ_1-42_ (5 and 20 μM) and SST (10 μM) for 24 h. Post-treatment, the cells were washed with phosphate-buffered saline (PBS) and incubated for 2 h at 37 °C in the presence of 300 μg/mL of methyl-thiazolyl diphenyl-tetrazolium bromide solution (Sigma) prepared in serum-free DMEM. The cells were subsequently washed in PBS, and the resulting formazan formed in the cells was dissolved in 200 μL of isopropanol for 15 min on a rotating shaker. The changes in color were analyzed using a spectrophotometer at a wavelength of 570 nm, with the background absorbance measured at 650 nm. The results are presented as percentage changes between the treated versus the control group.

### 2.3. Caspase/Apoptosis Activity Assay

The Aβ_1-42_ induced apoptosis in differentiated SH-SY5Y cells was analyzed using the Caspase-3/7 Green Apoptosis Assay kit (Essen Bioscience, Ann Arbor, MI, USA) following the manufacturer’s instructions. Briefly, SH-SY5Y cells were treated with Aβ_1-42_ (5 μM) alone or in combination with an increasing concentration of SST (0.4, 2, 10 μM) in the presence of a DNA intercalating dye NucView^TM^ 488 (Essen Bioscience). The resulting fluorescence was analyzed in the IncuCyte^TM^ live-cell imaging system (Essen Bioscience), and the Caspase-3/7 activity was assessed as an index of cells undergoing apoptosis using an IncuCyte basic analyzer (Essen Bioscience).

### 2.4. Live/Dead Cell Assay

The Aβ_1-42_-induced toxicity in the presence or absence of SST was also analyzed using a LIVE/DEAD Cell Vitality Assay (Thermo Fisher Scientific, Waltham, MA, USA), following the manufacturer’s instructions. The differentiated SH-SY5Y cells were treated with Aβ_1-42_ (5 μM) or SST (10 μM) alone or in combination for 24 h. Post-treatment, the cells were washed with PBS and collected in 0.05% trypsin-EDTA (Thermo Fisher Scientific). The cells were then re-suspended in 100 μL of PBS in the presence of C_12_-resazurin (20 ng/μL) and SYTOX dye (1 μM) and incubated for 15 min at 37 °C. Following incubation, the cells were immediately assessed on LSR II (BD Bioscience, San Jose, CA, USA) with excitation at 488 nm and emission at 530 and 570 nm, and analyzed using FlowJo workstation (BD Bioscience).

### 2.5. Western Blot Analysis

For the Western blot analysis, post-differentiation, control and treated SH-SY5Y cells were harvested using a lysis buffer (Cat# 9803; Cell Signaling) [40]. The total protein content of the cell lysate was determined using a Bradford assay, and whole-cell lysates (15 μg protein) were subjected to 10% SDS-polyacrylamide gel electrophoresis followed by transfer to nitrocellulose membrane. The membranes were blocked with 5% skim milk in TBS-T (Tris-buffered saline with 0.05% Tween-20) for 1 h at room temperature (RT) and immunoblotted overnight in the presence of respective rabbit polyclonal primary antibodies: C-terminal CRMP2 (1:1000; Cat # CP2161; ECM Bioscience, Versailles, KY, USA), Thr514-CRMP2 (1:1000, Cat# ab62478; Abcam, Cambridge, UK), Ser522-CRMP2 (1:1000, Cat# CP2191; ECM Bioscience), Thr555-CRMP2 (1:1000, Cat# CP2251; ECM Bioscience), SSTR2 (1:500, Cat# sc-25676; Santa Cruz Biotechnologies, Santa Cruz, CA, USA), SSTR4 (1:500, Cat# sc-25678; Santa Cruz Biotechnologies), Calpain I (1:500; Cat# 2556; Cell Signaling). Other antibodies used were mouse monoclonal CDK5 (1:2000; Cat# 05-364; Millipore) and rabbit monoclonal p35/25 (1:250; Cat# 64310; Cell Signaling). After incubation with the primary antibodies overnight, the membranes were washed in TBST and incubated for 1 h at RT with either horseradish peroxidase (HRP)-conjugated goat anti-mouse (1:2000) or goat anti-rabbit secondary antibodies (1:2000) (Jackson Lab). The membranes were washed in TBST and developed using a chemiluminescence detection kit (Millipore, Billerica, MA, USA) on Alpha Innotech FluorChem 8800. β-actin was used as a loading control. A densitometric analysis of protein expression levels was performed using ImageJ software.

### 2.6. Immunofluorescence Immunocytochemistry

The control and treated cells were fixed with 4% paraformaldehyde for 20 min and permeabilized with 0.1% Triton-X100 in PBS for 15 min at RT. Following three washes in PBS, the cells were blocked with 5% Normal Goat Serum (NGS) for 1 h at RT. The cells were then incubated with rabbit polyclonal primary antibody Ser522-CRMP2 (Cat# CP2191; ECM Bioscience) and mouse monoclonal βIII Tubulin (Cat# 801202; BioLegend) in 5% NGS overnight at 4 °C. Following the overnight incubation with the primary antibodies, the cells were washed with PBS and incubated with Alexa-conjugated secondary antibodies for 1 h at RT (1:200; Invitrogen). For nucleus visualization, the cells were incubated with Hoechst dye 33258 (0.5 μg/mL, Calbiochem, La Jolla, CA, USA) for 10 min at RT. The coverslips were then mounted onto the slides and photographed using a Zeiss LSM700 confocal microscope (Carl Zeiss, Oberkochen, Germany). Image panels were constructed using Carl Zeiss Zen software.

### 2.7. Agonist Treatment

SSTR2 and 4 specific non-peptide agonists (L-779976 and L-803087) were kindly provided by Dr S.P. Rohrer, Merck. Briefly, the differentiated SH-SY5Y cells were treated with SSTR specific agonists (3, 10, 30 nM) with or without Aβ for 24 h. Following treatment, the whole cell lysate prepared was processed to determine the expression levels and the activity of proteins of interest using Western blot analysis.

### 2.8. Fluo-4 Calcium Assay

The intracellular calcium levels were assessed using the Fluo-4 Direct^TM^ calcium assay kit (Invitrogen) following the manufacturer’s instructions. Briefly, the SH-SY5Y cells were plated onto a 96-well plate coated with Matrigel and differentiated with RA for up to 5 days. Following differentiation, the cells were incubated with an equal volume of 2 X Fluo-4 Direct^TM^ calcium reagents (including probenecid) at 37 °C for 60 min. Following the loading of the dye, the cells were treated with Aβ_1-42_ (5 or 20 μM) or SST (10 μM) alone and in a combination. The changes in the fluorescence intensity were measured (excitation at 494 nm and emission at 516 nm) in a spectrophotometer in a time-dependent manner for 50 cycles (20 s each). Untreated cells were used as internal control. The changes in absorbance are presented as a fold-difference between the treatment versus control (*n* = 3; each experiment represents an average of 3–6 independent readings).

### 2.9. Statistical Analysis

All results are presented as mean ± SD of a minimum of three independent experiments, as indicated. All statistical analyses have been performed in Graph Prism5.0. Student’s *t*-test, or one-way analysis of variance (ANOVA) was used as indicated. * *p* < 0.05 against control or Aβ_1-42_ treatment was taken into consideration as significant.

## 3. Results

### 3.1. SST Inhibits Aβ_1-42_-Induced Toxicity in Differentiated SH-SY5Y Cells

**Figure 1:**
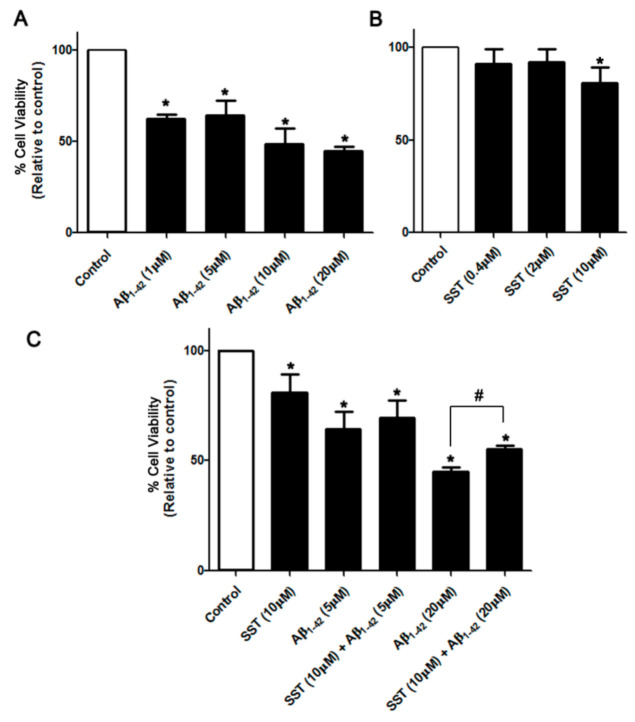
SST inhibits Aβ-induced cytotoxicity. Changes in cell survival following treatment with increasing concentrations of Aβ and SST alone or in combination were assessed by the MTT assay. (**A**) Aβ_1-42_ induced dose-dependent toxicity in differentiated SH-SY5Y cells with maximal toxicity observed at 20 μM of Aβ_1-42_. In contrast, SST displayed a marginal cytotoxic effect at higher doses only, without any significant effect at the lower concentrations (**B**). Cells treated with Aβ_1-42_ (5 and 20 μM) in combination with SST (10 μM) displayed enhanced cell viability when compared to Aβ_1-42_ alone (**C**). The data represent the mean ± SD of three independent experiments. * *p* < 0.05 against control; # against Aβ_1-42_ (20 μM).

To determine the cell viability of SH-SY5Y cells in response to Aβ_1-42_-induced toxicity, multiple approaches were applied. Initially, the overall cell metabolism was assessed using MTT assay as recently described [43]. As shown in Figure 1A, in response to increasing the concentration of Aβ_1-42_ (1, 5, 10 and 20 µM), differentiated SH-SY5Y cells exhibited dose-dependent toxicity in comparison to controls. At lower doses, SST displayed no significant effect on cell viability, whereas, at the higher dose (10 µM), SST produced a cytotoxic effect post 24 hr treatment (Figure 1B). However, differentiated cells treated with Aβ_1-42_ (5 and 20 µM) in combination with SST (10 µM) display enhanced cell viability when compared to Aβ_1-42_ alone (Figure 1C).

**Figure 2:**
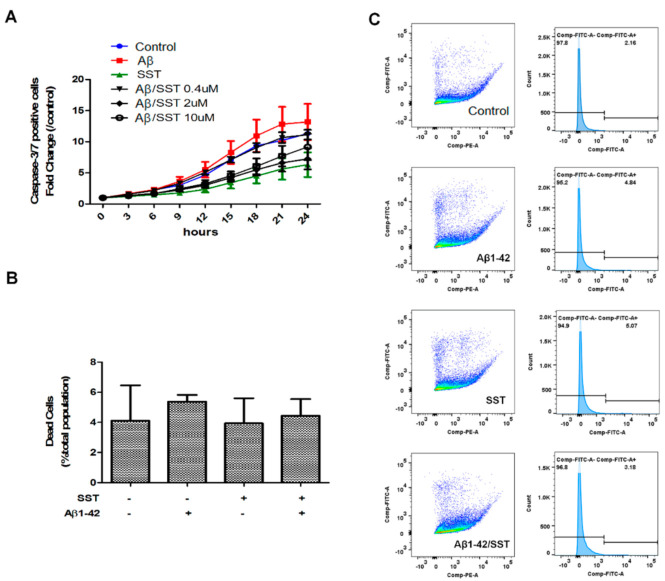
SST inhibits the Aβ-induced activation of apoptosis. (**A**) Apoptosis induction was assessed by measuring caspase-3/7 activity. Cells treated with Aβ (5 μM) alone displayed an elevation of caspase-3/7 activity, while cells treated with SST alone (10 μM) exhibited the lowest caspase-3/7 activity. Co-treatment of Aβ_1-42_ (5 μM) and SST resulted in reduced caspase-3/7 activity compared to the cells treated with Aβ_1-42_ alone. Data are shown as a fold-change against 0 h time point. (**B**) Cell viability assessed by a live/dead assay using metabolic activity and cell permeability as an index following treatment with Aβ (5 μM) and SST (10 μM) alone or in combination (**C**). Representative FACS data of C_12_-resazurin and SYTOX fluorescence intensity (dot plot) and FITC intensity distribution (histogram) displaying the distribution of cells based on viability. The cells were treated with Aβ (5 μM) and SST (10 μM) alone or in combination. The data represent the mean ± SD of three independent experiments.

Next, we assessed the effect of Aβ_1-42_ on cell viability by evaluating the activity level of caspase-3/7 as an index of apoptosis. As shown in Figure 2A, the SH-SY5Y cells treated with Aβ_1-42_ displayed an increase in basal caspase-3/7 activity that was significantly different when compared to the control. In contrast, the cells treated with SST alone displayed inhibition of caspase-3/7 activity. As shown in Figure 2A, SST in combination with Aβ_1-42_ displayed time- and concentration-dependent inhibition of caspase-3/7 activity when compared to the cells treated with Aβ_1-42_ alone. These results suggest that SST mediates the inhibition of Aβ-induced apoptosis in differentiated SH-SY5Y cells.

To determine the changes in metabolism as well as cell membrane integrity in response to the Aβ_1-42_-induced toxicity, a Live/Dead cell assay was performed in SH-SY5Y cells. Interestingly, the Live/Dead assay did not show significant changes in metabolic activity, which may be due to the metabolic demand of cells undergoing apoptosis (Figure 2B,C). However, when assessed strictly for the cell membrane integrity, the Live/Dead cell assay showed an increasing trend in cell permeability upon treatment with Aβ_1-42_ alone, albeit insignificantly, indicative of the toxic effect of Aβ (Figure 2B).

### 3.2. Somatostatin Downregulates the Phosphorylation of CRMP2 at the Ser522 Site

Previous studies have demonstrated that SST, when used in combination with neurite-promoting drugs, including nerve growth factor (NGF), brain-derived nerve growth factor (BDNF), or RA, increases the neurite outgrowth and promotes the differentiation of various cells, including SH-SY5Y cells [40,44]. It is well known that CRMP2 plays a critical role in mediating tubulin stability and neurite outgrowth [45]. However, whether SST-mediated neurite growth and elongation is directly associated with the suppression of CRMP2 phosphorylation in Aβ_1-42_-induced toxicity model is not well understood. Accordingly, we sought to examine whether SST attenuates the Aβ_1-42_-induced hyperphosphorylation of CRMP2 using Western blot analysis. Differentiated SH-SY5Y cells were treated with increasing concentrations of SST (0.4, 2 and 10 μM) in the presence of Aβ_1-42_ (5 μM). Vehicle treated cells or the cells treated with scrambled Aβ_42-1_ were considered as controls.

**Figure 3:**
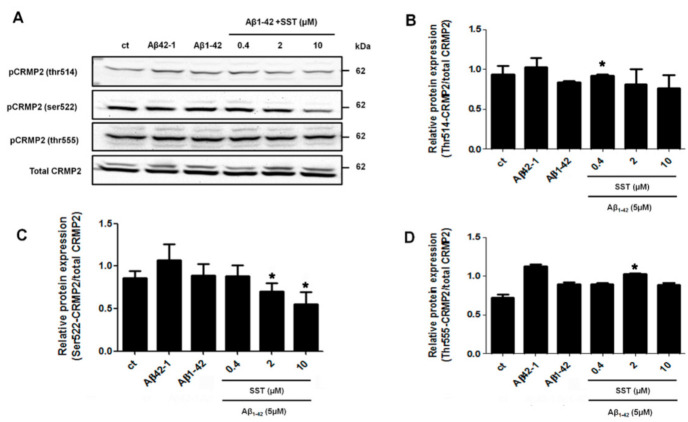
SST-mediated regulation of CRMP2 (Ser522) phosphorylation. (**A**). Representative Western blot showing the decreased phosphorylation level of Ser522-CRMP2 with increasing concentration of SST. Total CRMP2 was used as a loading control. B-D. Graphs represent the densitometry analysis of Western blot data shown in A for Thr514-CRMP2 (**B**), Ser522-CRMP2 (**C**), and Thr555-CRMP2 (**D**). Note that the Ser522-phosphorylation level is inhibited in a dose-dependent manner with increasing concentration of SST in the presence of Aβ. The data represent the mean ± SD of three independent experiments. * *p* < 0.05 against Aβ_1-42_ treated alone.

Furthermore, to determine changes in site-specific phosphorylation, three phosphorylation sites of CRMP2 that have been previously reported to be hyperphosphorylated in AD patients were selected (Thr514, Ser522 and Thr555) [46]. As shown in Figure 3, the phosphorylation levels of Thr514- or Thr555-CRMP2 did not show a dose-dependent response to any of the concentrations of SST in combination with Aβ_1-42_ (Figure 3, panels A, B, and D). Although the level of CRMP2 phosphorylation at site Ser522 was not relatively altered by Aβ_1-42_ alone, it was significantly downregulated in the presence of SST in a dose-dependent manner, with a maximal reduction in the presence of SST at 10 μM (Figure 3, panels A and C). Therefore, based on the cell viability assay and site-specific Thr522-CRMP2 phosphorylation, all subsequent experiments were performed using 10 μM of SST.

### 3.3. Somatostatin Inhibits the Activation of CRMP2 in the Presence of Aβ

**Figure 4:**
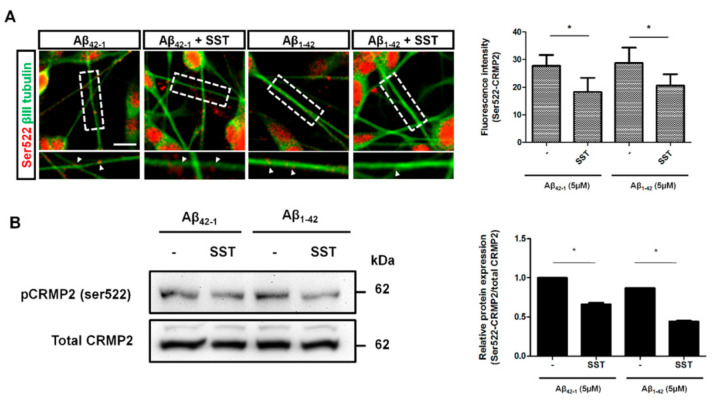
SST inhibits S522-CRMP2 phosphorylation. (**A**). Immunofluorescence staining image of Ser522-CRMP2 (red) co-stained with neuronal tubulin marker βIII-tubulin (green). The colocalization of Ser522-CRMP2 on neurites with βIII-tubulin is indicated (arrowhead; inset), and the average fluorescence intensity of S522-CRMP2 (in red) is shown on the bar graph. (**B**). Representative Western blot showing reduced phosphorylation at Ser522-CRMP2 in the presence of SST (bottom panel). The densitometry analysis of the Western blot is corroborated with a significant reduction in the phosphorylation level in the presence of SST in both Aβ_42-1_ and Aβ_1-42_ treated cells (upper panel). The data represent the mean ± SD of three independent experiments. * *p* < 0.05 against respective control; Scale bar = 20 µm.

To determine whether increased CRMP2 phosphorylation at Ser522 is associated with neurite formation, the subcellular distribution and colocalization of phosphorylated CRMP2 at Ser522 and neuronal tubulin marker βIII-tubulin was determined. In differentiated cells treated with scramble Aβ_42-1_, CRMP2-like immunoreactivity was confined primarily to the cell body, along with some punctuated staining in neurites (Figure 4A). The cells were mostly devoid of any colocalization and displayed no detectable changes in the presence of SST. Conversely, treatment with Aβ_1-42_ induced CRMP2 phosphorylation in neurites and showed colocalization with βIII-tubulin. However, following treatment with Aβ_1-42_ in combination with SST, CRMP2-like immunoreactivity was decreased, while the cells exhibited an increase in the expression of βIII-tubulin. 

To further validate whether CRMP2 phosphorylation at Ser522 in the presence of Aβ is abolished by SST, differentiated SH-SY5Y cells were treated with SST alone or in combination with Aβ_42-1_ or Aβ_1-42_, and the cell lysate prepared was processed for immunoblot analysis. As shown in Figure 4B, cells treated with SST displayed significant inhibition on Aβ_1-42_-mediated CRMP2 phosphorylation at Ser522 in comparison to cells treated with Aβ_42-1_. A quantitative analysis of the changes in CRMP2 phosphorylation (Ser522) was determined by a densitometric analysis (Figure 4B). These results suggest that SST suppresses the subcellular distribution of CRMP2 in SH-SY5Y cells and prompt the dissociation from βIII-tubulin in neurite formation.

### 3.4. SST Inhibits the Aβ_1-42_-Induced Over-Expression of SSTR4

**Figure 5:**
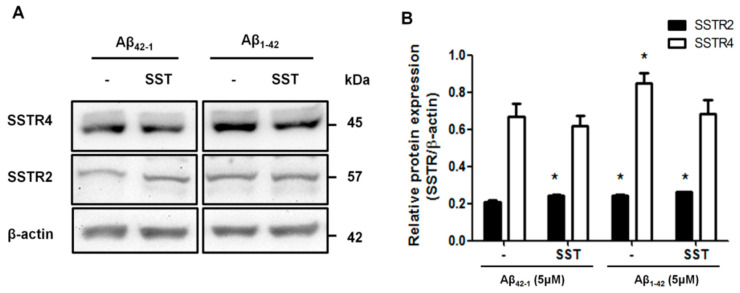
SST-induced changes in SSTR2 and SSTR4 expressions. (**A**). Representative Western blot showing the effect of SST in the expression of SSTR2 and 4. (**B**). The densitometry analysis of the Western blot shows that the treatment of cells with SST resulted in a significant increase in SSTR2 expression in the presence of either Aβ_42-1_ or Aβ_1-42_. SSTR4 expression increased following the treatment with Aβ_1-42_. In contrast, the co-treatment of cells with Aβ_1-42_ and SST resulted in the inhibition of Aβ_1-42_-induced upregulation of SSTR4. The data represent the mean ± SD of three independent experiments. * *p* < 0.05 against respective control.

The biological effects of SST are mediated by binding to five different receptor subtypes (SSTR1-5). We recently reported the role of SSTR2 and 4 in promoting the RA-induced neuronal differentiation of SH-SY5Y cells [40]. Here, accordingly, we monitored the changes in the expression of SSTR2 and 4 following treatment with either Aβ_1-42_ alone or in combination with SST. Scrambled Aβ_42-1_ was used as a control. SH-SY5Y cells were treated with Aβ_42-1_ (5 μM) and Aβ_1-42_ (5 μM) in the presence and absence of SST (10 μM) for 24 h. Post-treatment, cell lysates were collected and processed for immunoblot analyses for the expression of SSTR2 and 4. As shown in Figure 5, the cells treated with Aβ_42-1_ in the presence of SST exhibited an increase in SSTR2 expression without any discernible changes in SSTR4 expression. In contrast, the SH-SY5Y cells treated with Aβ_1-42_ displayed an increased expression of both SSTR2 and 4 when compared to the cells treated with Aβ_42-1_. In the cells treated with SST in combination with Aβ_1-42_, SSTR2 expression remained higher than Aβ_42-1_-treated cells but was comparable to the cells treated with Aβ_1-42_ alone. Interestingly, the cells treated with Aβ_1-42_ in combination with SST showed a significant reduction of SSTR4 expression when compared to cells treated with Aβ_1-42_ alone. These results indicate SST-induced changes in subtype-specific receptor internalization, desensitization, and degradation.

### 3.5. SSTR-Subtypes-Mediated Changes in CRMP2 Phosphorylation

**Figure 6:**
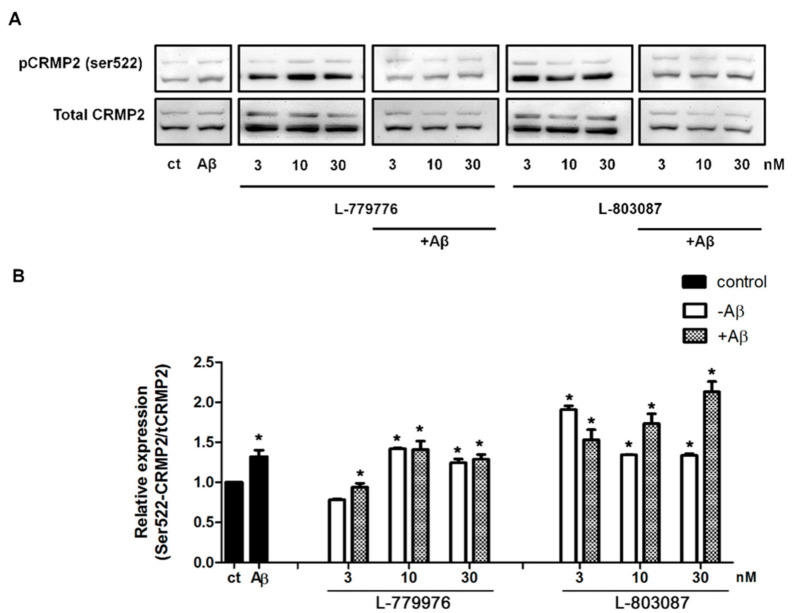
SSTR specific agonist effect on Ser522-CRMP2 phosphorylation. (**A**). Representative Western blot showing changes in the level of S522-CRMP2 phosphorylation in cells, following treatment with increasing concentrations of SSTR2 and SSTR4-specific agonist (3, 10, 30 nM) in the presence or absence of Aβ_1-42_ (5 μM). (**B**). The densitometry analysis of Western blot shows a significant inhibition of Ser522 phosphorylation upon treatment with L-779976 (3 nM) in the absence or presence of Aβ_1-42_. L-779976 (10 and 30 nM) alone, which resulted in the moderate elevation of phosphorylation at the Ser522 site compared to the untreated control. In the presence of Aβ_1-42_, L-779976 (10 and 30 nM) displayed moderate changes when compared to the cells treated with Aβ_1-42_. In the presence of Aβ_1-42_, L-803087 induced a dose-dependent increase of Ser522 phosphorylation, resulting in the highest level of expression at 30 nM treatment of L-803087. The data represent the mean ± SD of three independent experiments. * *p* < 0.05 against respective control.

To determine which receptor subtype is involved in the SST-mediated inhibition of CRMP2 activation, differentiated SH-SY5Y cells were treated with SSTR2 and 4 specific agonists alone or in the presence of Aβ for 24 hr. Post-treatment, cell lysates collected from controls and treated cells were processed for Western blot analysis to assess CRMP2 phosphorylation. As shown in Figure 6A, in comparison to the control, CRMP2 phosphorylation increased significantly in cells treated with Aβ_1-42_. Receptor agonists induced concentration-dependent changes on CRMP2 phosphorylation in a receptor-specific manner. As shown in Figure 6A, in the absence of Aβ_1-42_, at the lowest concentration (3 nM), SSTR2-specific agonist (L-779976) inhibits CRMP2 phosphorylation at Ser522, whereas at higher concentrations (10, 30 nM), a moderate increase in CRMP2 phosphorylation was observed. The differentiated SH-SY5Y cells treated with SSTR2 agonist (3 nM) in the presence of Aβ_1-42_ displayed inhibition of CRMP2 phosphorylation when compared to the cells treated with Aβ_1-42_ alone. However, in the presence of Aβ_1-42_ and SSTR2 agonist at higher concentrations (10 and 30 nM), no significant change in CRMP2 phosphorylation was observed when compared to Aβ_1-42_ treatment alone. Notably, a higher concentration of SSTR2 agonist displayed no apparent difference in the levels of CRMP2 phosphorylation with or without Aβ_1-42_.

As shown in Figure 6A, differentiated SH-SY5Y cells treated with SSTR4 agonist (L-803087) displayed significantly higher CRMP2 phosphorylation in comparison to controls. However, such enhanced status of CRMP2 phosphorylation was relatively higher at a lower concentration (3 nM), in contrast to a higher concentration, without any distinguishable difference between 10 and 30 nM. Next, we determined whether Aβ_1-42_ activated CRMP2 phosphorylation is suppressed in the presence of SSTR4 agonist. As shown in Figure 6B, the status of CRMP2 phosphorylation in cells treated with SSTR4 agonist in combination with Aβ_1-42_ exhibited a concentration-dependent increase that was significantly higher than both controls and cells treated with Aβ_1-42_ alone.

### 3.6. Somatostatin-Mediated Inhibition of Ser522-CRMP2 is Regulated Through the Calcium Pathway

**Figure 7:**
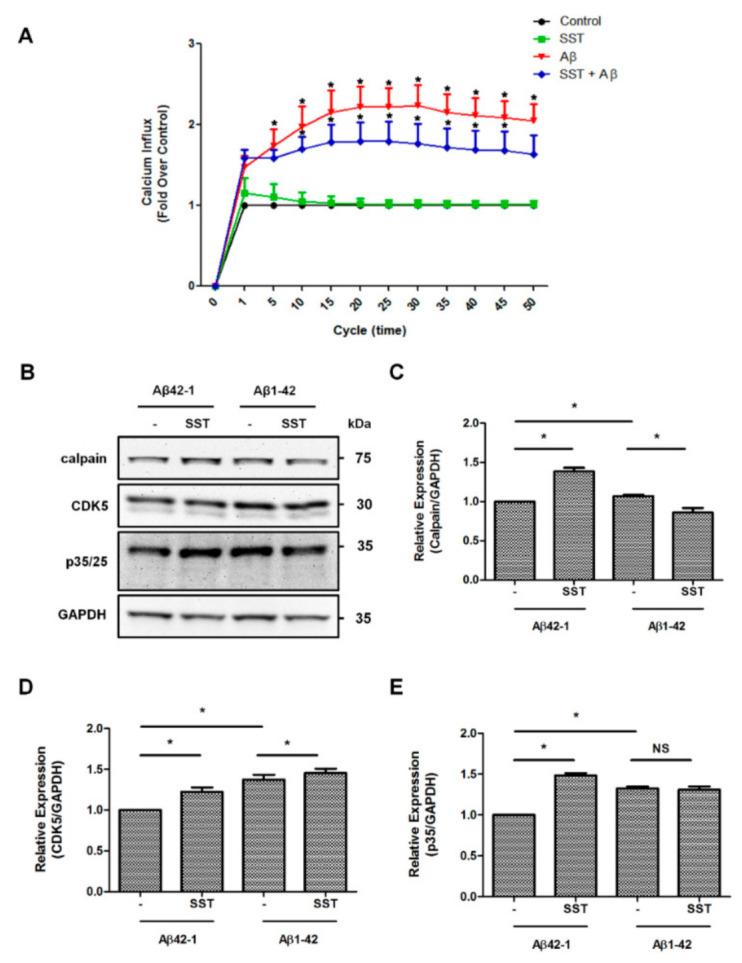
SST-mediated effects on calcium signaling and downstream mediators. (**A**). The intracellular level of Ca^2+^ was assessed using the Fluo-4 calcium indicator. Cells treated with Aβ_1-42_ resulted in an increased Ca^2+^ influx compared to the SST or untreated control. Cells treated with Aβ_1-42_ in the presence of SST resulted in a noticeable inhibition of the Ca^2+^ influx compared to the cells treated with Aβ_1-42_ alone. B. Representative Western blot displaying the changes in the expression of calpain, CDK5, and p35/25 expression in cells treated with Aβ_1-42_ in the presence or absence of SST. (**C**–**E**). Histograms represent the densitometry analysis of the Western blot shown in (**B**). Data represent the mean ± SD of three independent experiments. * *p* < 0.05 against respective control, Two-way ANOVA with Bonferroni post-hoc tests.

Increased intracellular Ca^2+^ accumulation is a well-documented mechanism of Aβ-mediated toxicity via inducing calpain activity, over-activation of CDK5, and hyper-phosphorylation of CRMP2 at Ser522, leading to the disassembly of the CRMP2 complex. Previous studies have suggested that SST inhibits Ca^2+^ by binding to SSTR2 [47,48,49]. To assess whether SST inhibits Aβ induced an increase in the Ca^2+^ influx, and the intracellular Ca^2+^ content was monitored using Fluo-4 in RA differentiated SH-SY5Y cells. In the cells treated with SST alone (10 µM), the intracellular Ca^2+^ level was comparable to the control. The cells treated with Aβ_1-42_ alone (5 µM) had no significant effect on intracellular Ca^2+^ levels at early time points (data not shown), whereas treatment with Aβ_1-42_ alone at a higher concentration of 20 µM induced a time-dependent increase in intracellular Ca^2+^ level within a short treatment duration (Figure 7A). The intracellular Ca^2+^ influx was suppressed and maintained at a lower level in cells treated with Aβ_1-42_ (20 µM) in combination with SST (10 µM) when compared to Aβ_1-42_ alone (Figure 7A). These results indicate that SST potentially inhibits an Aβ_1-42_-induced increase in the Ca^2+^ influx and supports possible mechanisms of SST-mediated neuroprotection in Aβ-induced toxicity.

Whether SST-mediated changes in the intracellular Ca^2+^ affected resulted in changes in calpain expression and CDK5 activity and their downstream p35/25 expression is not known. As shown in Figure 7B–E, differentiated SH-SY5Y cells treated with Aβ_42-1_ in combination with SST showed a significant increase in calpain expression in comparison to Aβ_42-1_ alone. The calpain expression in differentiated SH-SY5Y cells upon treatment with Aβ_1-42_ alone was not changed as compared to scramble. However, cells treated with Aβ_1-42_ in combination with SST displayed a significant inhibition of calpain expression in comparison to the cells treated with Aβ_1-42_ alone (Figure 7C). The CDK5 expression was also increased in the presence of SST and Aβ_42-1_ in combination when compared to the cells treated with Aβ_42-1_ (Figure 7D). In particular, the cells treated with Aβ_1-42_ and SST together also resulted in a significant increase in CDK5 expression compared to the cells treated with Aβ_1-42_ alone. Interestingly, such changes in calpain expression did not translate into changes in p35 expression. Instead, p35 expression increased significantly in the presence of SST in combination with Aβ_42-1_ as well as with Aβ_1-42_ alone or in combination with SST (Figure 7E). Taken together, in differentiated SH-SY5Y cells, these events are supposed to be interconnected but function independently.

## 4. Discussion

We recently described the role of SST in RA-induced neurite growth in SH-SY5Y cells and established a possible interaction with the changes in MAP2/Tau and TUJ1, as well as an ERK1/2 signaling pathway. We also uncovered that the cells displaying colocalization between SST and TUJ1 exhibited a more extended neurite growth than cells devoid of colocalization [40]. The intact neurite formation is essential for a normal neuronal function. In contrast, disrupted neurite organizations are often observed in neurological diseases, including AD, and are associated with impaired cognitive function and memory loss. Whether SST is involved in improving neurite outgrowth and maintaining neuronal integrity in Aβ-induced neurotoxicity is not known. In the present study, using differentiated SH-SY5Y cells, we describe the role of SST in Aβ-induced toxicity and the molecular determinants, including CRMP2, Ca^2+^ influx, CDK5, calpain, and P35/25, that might be associated with neurite outgrowth and cell viability. We demonstrate that SST improves cell viability and inhibits Aβ activated caspase 3/7 activity. We did not observe significant changes in metabolic activity as a proxy for Aβ-induced toxicity, and this might require higher concentrations of Aβ [50,51]. Furthermore, SST downregulates the influx of calcium level, which plays a pivotal role in the CDK5 activity. Our data suggest that SST mediates changes in CRMP2 phosphorylation and Aβ_1-42_-induced toxicity via the regulation of calcium in differentiated SH-SY5Y cells. This newly discovered mechanism might be involved in improving microtubules’ organization and neurite outgrowth in AD pathogenesis.

Amongst the neuropeptides studied to date, SST is one of the most significant peptides that changes during the onset and progression of AD, with a consistent reduction in both the cerebrospinal fluid and brain tissues of AD patients [52,53,54,55,56,57,58,59]. We have previously reported the neuroprotective role of SST against various neurotoxic insults, such as pro-inflammatory lipopolysaccharide and Aβ_1-42_ in a human cerebral micro-vessel cell line (hCMEC/D3), cultured cortical neurons, and cultured striatal neurons, as well as QUIN- and NMDA-induced excitotoxicity and cell death [43,60,61,62,63]. An intracerebroventricular (i.c.v) infusion of Aβ in rats led to the significant reduction of SST-positive neurons in various brain regions, including the hippocampus and the temporal and frontoparietal cortex [64,65,66,67]. Furthermore, studies have also shown colocalization between the somatostatinergic-neurons and Aβ plaques in brain regions, including the amygdala, cortex, and hippocampus, of AD patients [68,69]. Saito et al. reported that the activity of a potent inhibitor of Aβ accumulation, neprilysin, was elevated following the introduction of SST, resulting in a subsequent reduction of Aβ aggregation [70]. Consistent with these observations, in the present study, the SST-induced amelioration of the toxic effect of Aβ was corroborated via various toxicity assays, including MTT, caspase-3/7 activity assay, and LIVE/DEAD toxicity assay. Collectively, these findings suggest a significant neuroprotective role of SST against Aβ-induced toxicity. 

The impaired CRMP2 expression or activity may lead to a significant disruption in the overall neurite structure and a decline in cognitive function. CRMP2 is associated with various characteristics of neurite homeostasis, such as formation, outgrowth, and guidance, as well as maintaining the proper microtubule assembly by binding to the microtubule heterodimers and inducing polymerization while directly regulating tubulin GTPase activity [13,21,71,72,73]. The hyperphosphorylation of CRMP2 has been observed in NFTs as well as in the soluble fragments of the brain tissues derived from AD patients [2,74]. Furthermore, transgenic mouse models of AD, including (PSEN1 (M146V) KI, Thy1.2-AβPP (swe) and triple (PSEN1 (M146V) KI, Thy1.2- AβPP (swe), and Thy1.2-tau (P301L), exhibit a significant increase in CRMP2 phosphorylation in the hippocampus and cortex [2]. On the other hand, other transgenic mouse models of AD, such as Tg2576, P301L, or P301s tau, fail to show an increase in CRMP2 phosphorylation, suggesting that the combination of AβPP and PSEN1 mutation may be a prerequisite for dysfunctional CRMP2 phosphorylation. Consistent with these studies and in support of SST-mediated neuroprotective and neurite outgrowth promoting effects, we observed here that SST downregulated CRMP2 hyper-phosphorylation in the presence of Aβ_1-42_. Reduced CRMP2 phosphorylation, along with the increased expression of βIII-tubulin and its dissociation from CRMP2 in neurites upon treatment with SST, is an indication that tubulin is a prerequisite in the neurite elongation. It was not surprising to note that no significant elevation in CRMP2- Ser522 phosphorylation levels in SH-SY5Y cells was observed following treatment with Aβ_1-42_ in our study. A previous study has reported that the phosphorylation of CRMP2 at the T555 site was significantly elevated in the presence of Aβ_1-40_ in SH-SY5Y cells. The study reported no such changes at Thr514 and Ser522 sites and linked such variations to the Aβ species-dependent mechanism [75]. However, despite the differences in the CRMP2 phosphorylation levels, both Aβ_1-40_ and Aβ_1-42_ potentially impacted neurite length and elicit similar cellular outcomes [75]. Therefore, the role of Aβ_1-42_, Aβ_1-40_, or Aβ_25-35_ on the phosphorylation of various CRMP2 sites could be potentially explored in future studies.

Increased activation of CRMP2 in the presence of SSTR2 and 4 specific agonists is surprising and warrants future research. We have previously shown that both SSTR2 and 4 internalize in response to ligand binding [76,77]. Our past studies have shown that SSTR2 exists predominantly as homodimers on the cell surface, whereas SSTR4 exists as both monomers and homodimers [76,77]. The inhibition of CRMP2 phosphorylation at this lower dose of SSTR2 agonist suggests that SSTR2 internalization is not prompted at this concentration, but triggered at a higher concentration. Moreover, in the presence of SSTR4 agonist, the receptor internalization is expected at all the concentrations used and followed by degradation, which may account for CRMP2 phosphorylation, which may be even higher in the presence of Aβ. We have previously demonstrated that SSTR2 and SSTR4 exist as homo- and heterodimers on the cell surface, whereas agonist treatment leads to changes in the receptor dimerization and enhanced internalization [76,77]. Consistent with these observations, it is highly possible that the dissociation of SSTR2 and 4 homo- and heteromeric complexes at the cell surface in response to receptor activation resulted in enhanced CRMP2 phosphorylation.

Previous studies have shown increased phosphorylation of CRMP2 by CDK5 and GSK3β in AD patients when compared to the age-matched controls [2]. CDK5 is a serine/threonine kinase that is activated upon association with its substrate p35 or p39. The abnormal CDK5 expression or activity has been closely associated with neurotoxicity in various neurodegenerative diseases, including AD, HIV neurotoxicity, and prion-related encephalopathies [37,78,79]. Furthermore, the disruptions in intracellular calcium homeostasis have also been associated with the onset and progression of AD and other amyloidogenic diseases, such as Parkinson’s disease [80,81,82]. Various mechanisms have been suggested for the Aβ-mediated increase in calcium influx, including the disruption of lipid integrity [83], the formation of cation-selective channels by Aβ [81,84], or the activation of selective cell surface receptors to calcium [80,85,86]. These studies further emphasize that the Aβ-induced increase in calcium influx is not solely dependent on one particular pathway, but mediated through a complex network. In particular, the excess Ca^2+^ influx in the presence of Aβ leads to the calpain-mediated truncation of CDK5 substrate p35 into the much more stable form of p25, leading to the prolonged activation of CDK5, followed by the hyper-phosphorylation of downstream mediators such as CRMP2 [35,87,88].

SST through SSTR2 is known to inhibit the Ca^2+^ influx [47,48,49]. In agreement with previous studies, we found an increased expression of SSTR2 upon treatment with SST in the presence or absence of Aβ, supporting SSTR2 as the essential receptor involved in the SST-mediated inhibition of Ca^2+^ influx. Although the observed inhibitory changes in Ca^2+^ influx were not significantly different between Aβ_1-42_ alone or in combination with SST-14 due to higher deviations, a similar trend was observed in all the experiments performed. We predict that the assay’s high sensitivity and changes in the baseline due to experimental variability might be the reason for such observation. Furthermore, we have also observed a significant inhibition of calpain expression in cells co-treated with Aβ and SST compared to the cells treated with Aβ alone. This inhibition of calpain expression by SST did not result in the inhibition of CDK5 expression. Still, it resulted in a significant decrease of CRMP2 phosphorylation at Ser522, suggesting that SST might inhibit the hyperphosphorylation of CRMP2 by interfering with Ca^2+^ homeostasis. Furthermore, as the CDK5-mediated phosphorylation of downstream targets such as CRMP2 depends on the activity rather than the expression level of CDK5, a significant change in CDK5 activity mediated by SST is conceivable, and future studies are warranted in this direction.

The activation of CRMP2 (Ser522) upon treatment with SSTR2 and 4 specific agonists, in contrast to SST-mediated attenuation, is intriguing. However, the molecular mechanism associated with such contradicting results is not known. Whether the SST-mediated suppression of CRMP2 (Ser522) phosphorylation is due to the direct or indirect activation of multiple SSTR subtypes warrants further research. Furthermore, it is possible that unlike SST, which is highly associated with the Ca^2+^ uptake, specific SSTRs may work independently of the calcium pathway and via the modulation of downstream signaling pathways. Importantly, the role of other CRMP phosphorylation sites and isoforms, specifically CRMP5, cannot be avoided from the discussion. Previous studies have shown that CRMP5 inhibits neurite outgrowth and antagonizes CRMP2-mediated axonal and dendrite growth [89]. It is highly possible that SSTR2 and 4 agonists might inhibit CRMP5, resulting in enhanced CRMP2 phosphorylation.

## 5. Conclusions

In conclusion, the findings from the current study elucidate the mechanistic regulatory role of SST in intracellular calcium homeostasis, CRMP2 phosphorylation, and neurite formation and integrity. These observations corroborate the neuroprotective role of SST in neurotoxicity and neurodegenerative diseases by suggesting a novel mode of action. Furthermore, as disrupted calcium homeostasis is restricted to the neurodegenerative disease, the effective regulation of calcium levels by SST may have significant therapeutic applicability.

## Data Availability

The data supporting the findings of this study are available within the article.
